# Consensus terminology for preclinical phases of psoriatic arthritis for use in research studies: results from a Delphi consensus study

**DOI:** 10.1038/s41584-021-00578-2

**Published:** 2021-02-15

**Authors:** Lourdes M. Perez-Chada, Rebecca H. Haberman, Vinod Chandran, Cheryl F. Rosen, Christopher Ritchlin, Lihi Eder, Philip Mease, Soumya Reddy, Alexis Ogdie, Joseph F. Merola, Jose U. Scher

**Affiliations:** 1 Department of Dermatology, Harvard Medical School, Brigham and Women’s Hospital, Boston, MA USA; 2 Department of Medicine, Division of Rheumatology, New York University Langone Health, New York, NY USA; 3 Department of Medicine, Division of Rheumatology, University of Toronto, Toronto, ON Canada; 4 Psoriatic Arthritis Program, Centre for Prognosis Studies in the Rheumatic Diseases, Krembil Research Institute, University Health Network, Toronto, ON Canada; 5 Division of Dermatology, Toronto Western Hospital, University of Toronto, Toronto, ON Canada; 6 Allergy, Immunology, and Rheumatology Division, University of Rochester Medical School, Rochester, NY USA; 7 Women’s College Research Institute, Women’s College Hospital, University of Toronto, Toronto, ON Canada; 8 Seattle Rheumatology Associates, Swedish Medical Center and Providence St, Joseph Health, Seattle, WA USA; 9 University of Washington School of Medicine, Seattle, WA USA; 10 Department of Medicine, Division of Rheumatology, Center for Clinical Epidemiology and Biostatistics, Perelman School of Medicine, University of Pennsylvania, Philadelphia, PA USA; 11 Department of Medicine, Division of Rheumatology, Harvard Medical School, Brigham and Women’s Hospital, Boston, MA USA

**Keywords:** Psoriatic arthritis, Clinical trial design

## Abstract

The concept of psoriatic arthritis (PsA) prevention is gaining increased interest owing to the physical limitation, poor quality of life and low remission rates that are achieved with current therapies for PsA. The psoriasis-to-PsA transition offers a unique opportunity to identify individuals at increased risk of developing PsA and to implement preventive strategies. However, identifying individuals at increased risk of developing PsA is challenging as there is no consensus on how this population should be defined. This Consensus Statement puts forward recommended terminology from the Psoriasis and Psoriatic Arthritis Clinics Multicenter Advancement Network (PPACMAN) for defining specific subgroups of individuals during the preclinical and early clinical phases of PsA to be used in research studies. Following a three-round Delphi process, consensus was reached for three terms and definitions: ‘increased risk for PsA’, ‘psoriasis with asymptomatic synovio-entheseal imaging abnormalities’ and ‘psoriasis with musculoskeletal symptoms not explained by other diagnosis’. These terms and their definitions will enable improved identification and standardization of study populations in clinical research. In the future, as increasing evidence emerges regarding the molecular and clinical features of the psoriasis-to-PsA continuum, these terms and definitions will be further refined and updated.

## Abstract (web summary)

In this Consensus Statement, an expert panel from the Psoriasis and Psoriatic Arthritis Clinics Multicenter Advancement Network (PPACMAN) recommends terminology for defining specific subgroups of individuals during the preclinical and early clinical phases of psoriatic arthritis to be used in research studies.

## Introduction

Psoriatic arthritis (PsA) is a chronic, immune-mediated inflammatory disease, characterized by both skin and joint involvement. Synovio-entheseal involvement is present in up to 30% of those with psoriasis^1,2^, and individuals with psoriasis progress to PsA at a rate of up to 3% per year^3^. PsA can lead to joint erosions and deformities^4^, as well as to decreased quality of life^5^, high levels of psychosocial stress^6^ and increased rates of comorbidities, unemployment, absenteeism and productivity loss^7^. Despite this burden, PsA remains both underdiagnosed and undertreated, even within dermatology practices^8,9^. The current challenges in diagnosing and treating PsA produce a considerable gap in the care of patients with psoriatic disease, given that a delay in treatment of as little as 6 months can lead to worse disease outcomes^10,11^.

Highly effective treatment strategies are a major unmet need in PsA, and various interventions have been envisioned, including innovative therapeutic targets, combination therapies or potentially preventive measures. These last have become a focus in the field of PsA research and will be aimed at defining, predicting and, ultimately, preventing synovio-entheseal inflammation. To this end, it will be necessary to identify individuals at increased risk of developing PsA and to characterize clinical and molecular features that are specific to preclinical stages of disease. Distinguishing these high-risk individuals will help to shape the development, design and implementation of PsA prevention trials. In addition, it will enable improved screening, earlier diagnosis, timely treatment initiation and, eventually, should improve overall disease outcomes.

The Psoriasis and Psoriatic Arthritis Clinics Multicenter Advancement Network (PPACMAN) is an international non-profit organization that aims to “optimize the clinical care of patients with psoriatic disease through multidisciplinary collaboration, education and innovative research”^12^. Within PPACMAN, the Preventing Arthritis in a Multicenter Psoriasis At-Risk Population (PAMPA) study group was established to understand the clinical, genetic, environmental and immune events that occur during the natural history of the psoriasis-to-PsA transition^13^. To facilitate research focused on the preclinical and early clinical phases of PsA, the PAMPA study group conducted a consensus-building exercise to agree on common terminology related to the preclinical phases of PsA for use in clinical trials and translational research. The development of standardized nomenclature and common definitions to be used exclusively for research in this area should help in the recruitment of well-defined, homogeneous cohorts of patients and enable comparison across future trials and experimental therapeutic studies. This exercise emulates the efforts taken to create the EULAR recommendations for terminology for those at risk of rheumatoid arthritis (RA)^14^. In this Consensus Statement, we describe the process and results of a consensus-building exercise to develop nomenclature for preclinical PsA that can be integrated into future research studies.

## Methods

### Scientific committee

The consensus exercise was led and designed by the PAMPA study group and the PPACMAN steering committee, composed of methodologists, as well as dermatologists and rheumatologists who are experts in psoriatic disease.

### Overview of study design and methods

In this study, an online Delphi process was used that included international experts in psoriatic disease to achieve consensus on the terminology related to the preclinical phases of PsA for research purposes. The Delphi method is an iterative series of structured rounds that surveys experts until group consensus is reached. This method lends itself well to an online format, which enables a larger number of international experts to participate in the survey and avoids any strong influences from a small number of individuals or from standards of practice in certain countries. The Delphi method is used widely in health-care-related research that relies on expert opinion^15,16^; however, some limitations must be acknowledged. The Delphi methodology has been criticized for a possible lack of sufficient details about the background information provided to participants, differing response rates for all rounds, lack of formal feedback between rounds and lack of ability to discuss disagreement directly^17,18^. This Delphi exercise was therefore carefully designed to provide sufficient background detail and feedback for each round and to provide intermittent opportunities for direct discussion. For this study, a pre-Delphi exercise and three rounds of Delphi surveys were used.

#### Pre-Delphi exercise

Prior to the Delphi development, experts in psoriasis and PsA (*n* = 28) were queried via e-mail regarding terms and definitions of the phases that individuals with psoriasis might go through prior to PsA development. This group of experts was recruited from PPACMAN and also included members of the Group for Research and Assessment of Psoriasis and Psoriatic Arthritis (GRAPPA), an international non-profit organization, and the National Psoriasis Foundation (NPF), a non-profit organization from the USA. On the basis of these results, preliminary terms and definitions were drafted and presented at the PPACMAN 2018 Annual Meeting^19^. After an introductory session, four breakout workshop sessions took place in which attendees suggested changes and provided open-ended opinions about the terms and definitions proposed in the form of small group discussions. These workshop sessions were followed by a plenary session in which the outcomes of each breakout workshop were summarized, and culminated with a voting exercise via an anonymous automated response system. This meeting included dermatologists (*n* = 8), rheumatologists (*n* = 13), rheumatologist–dermatologists (*n* = 2) and industry representatives (*n* = 17). Notably, the industry representatives met in a separate breakout session and did not participate in the voting process to avoid the introduction of bias into the study findings. Further details on the pre-Delphi session can be found in the Supplementary Information. The collected input and results were used to inform the development of the subsequent Delphi survey.

#### Delphi process

The Delphi survey was designed using feedback from the pre-Delphi exercise. The survey was created and distributed using the New York University REDCap software^20^ to 45 international experts in psoriasis and PsA who were selected by the scientific committee. As with the pre-Delphi exercise, experts were recruited from PPACMAN and included members of GRAPPA and the NPF, and all members who participated in the pre-Delphi exercise were invited to participate in the Delphi exercise. These experts had an average of over 18 years of experience in their fields (Table 1). No members of industry were invited to participate. All answers were anonymous, and participants were asked to vote on and rank their preferred terms and definitions for describing populations of individuals with preclinical PsA. Space for free-form comments was also provided for each question. In all rounds, participants were provided with the results and discussion points generated in the previous rounds, as well as links to published literature to provide background information related to the voting topics^3,13^. Results from the first Delphi round were discussed at the PPACMAN meeting adjacent to the 2019 Annual Meeting of GRAPPA by multiple stakeholders. Consensus for multiple choice and ranking questions was defined a priori as ≥70%. For questions that use a visual analogue scale (VAS) ranging from 0 mm (should not be considered) to 100 mm (should definitely be considered), items were retained if the median score was >70 mm. If consensus was not reached, the question was carried through to the next round; however, Delphi items rated on a VAS that had a median score of <60 mm were removed.

**Table 1:** Numbers and demographics of participants in each Delphi round

Demographic	First round (*n* = 29)	Second round (*n* = 33)	Third round (*n* = 35)
***Type of participant***			
Dermatologist	5 (17.2%)	6 (18.2%)	10 (28.6%)
Rheumatologist	20 (69.1%)	22 (66.7%)	22 (62.9%)
Rheumatologist–dermatologist	3 (10.3%)	3 (9.1%)	3 (8.6%)
Non-clinician researcher	1 (3.4%)	2 (6.1%)	0 (0.0%)
***Location of participant***			
USA	18 (62.1%)	20 (60.6%)	24 (68.6%)
Europe	7 (24.1%)	9 (27.3%)	7 (20.0%)
Canada	4 (13.8%)	4 (12.1%)	4 (11.4%)
***Gender of participant***			
Female	12 (41.4%)	15 (45.5%)	16 (45.7%)
Male	17 (58.6%)	18 (54.5%)	19 (54.3%)
***Amount of experience of participants***			
Mean number of years (s.d.)	18.70 (10.47)	17.36 (10.42)	18.86 (11.24)
***Type of experience of participants***			
Academic clinicians	25 (86.2%)	26 (78.8%)	28 (80.0%)
Clinicians who work privately	1 (3.4%)	3 (9.1%)	3 (8.6%)
Both	3 (10.3%)	4 (12.1%)	4 (11.4%)

#### Data analysis

Descriptive statistics were used to report the voting results. Continuous data are presented as medians and interquartile range unless otherwise noted.

## Results

### Pre-Delphi exercise

At the 2018 PPACMAN Annual Meeting, stakeholders were presented with preliminary terms and definitions that were informed by the input collected from the initial survey distributed by e-mail. Expanded discussions and voting followed, but no consensus was reached. Further details of the pre-Delphi exercise are provided in the Supplementary Information.

### Delphi exercise

Round 1 of the Delphi exercise received 29 responses (response rate 64.4%), round 2 received 33 responses (response rate 73.3%) and round 3 received 35 responses (response rate 77.7%). Although the invited participants varied with regard to demographics and experience, the respondents were rheumatologists, dermatologists and rheumatologist–dermatologists who were mostly academic clinicians and/or researchers (Table 1). The original terms and definitions presented for voting in the Delphi exercise are provided in the Supplementary Information. Box 1 shows the final terms and definitions proposed after consensus was reached.

Box 1 PAMPA consensus terminology for preclinical phases of PsA According to the proposed terminology, in prospective research studies, individuals would be described in the following ways:

**Individuals with psoriasis at increased risk for PsA**

Any individual with psoriasis and one or more risk factors for progression to psoriatic arthritis (PsA).

- Risk factors include obesity, the presence of arthralgia, severe psoriasis, a history of uveitis, nail psoriasis, scalp psoriasis, having a first-degree relative with PsA and any associated gene (such as HLA-B*08, HLA-B*27, HLA-B*38 or HLA-B*39).
- Can be combined with either of the other two terms.

**Individuals with psoriasis and asymptomatic synovio-entheseal imaging abnormalities**

Any individual with psoriasis and imaging evidence of synovio-entheseal abnormalities that is not associated with clinical signs or symptoms.

- Imaging modalities include MRI for axial disease, MRI for peripheral arthritis, ultrasonography for peripheral arthritis, ultrasonography for enthesitis and plain radiography for peripheral arthritis. Specific MRI findings used to define imaging abnormalities include enthesitis, bone marrow oedema, synovitis, tendonitis, bone erosions and new bone formation. Specific ultrasonography findings used to define imaging abnormalities include enthesitis, synovitis, tendonitis and bone erosions.
- Can be combined with ‘individuals with psoriasis at increased risk for PsA’; for example, an individual with psoriasis might have uveitis (a risk factor for PsA) and have asymptomatic enthesitis defined by ultrasonography.
- Cannot be combined with ‘individuals with psoriasis and musculoskeletal symptoms not explained by other diagnosis’.

**Individuals with psoriasis and musculoskeletal symptoms not explained by other diagnosis**

Any individual with psoriasis and heel pain, stiffness and/or arthralgia not explained by another diagnosis.

- Can be combined with ‘individuals with psoriasis at increased risk for PsA’; for example, an individual with psoriasis might have uveitis (a risk factor for PsA) and have heel pain that is not explained by another diagnosis.
- Cannot be combined with ‘individuals with psoriasis and asymptomatic synovio-entheseal imaging abnormalities’.

### Terms and definitions

#### Individuals with psoriasis at increased risk for PsA

During round 1 of the Delphi exercise, 80% of the panellists agreed on the term ‘increased risk for PsA’, and 86% voted that this term defines a meaningful subgroup for future research studies. Other terms proposed included ‘at risk’, ‘high risk’, ‘higher risk’ and ‘elevated risk’. The term ‘at risk’ for PsA was not favoured by participants, who noted that any patient with psoriasis has the potential to develop PsA. During round 1, consensus for the definition was not reached.

In round 2 of the Delphi exercise, the definition of ‘any individual with psoriasis and one or more risk factors for progression to PsA’ reached consensus with 84.4% agreement. The alternative definition proposed was ‘any individual with psoriasis and one or more risk factors for progression to synovio-entheseal disease’. Participants noted that the term synovio-entheseal disease might not be commonly used, particularly by dermatologists.

Regarding which risk factors for progression from psoriasis to PsA should be considered, obesity, the presence of arthralgia, severe psoriasis, a history of uveitis, nail psoriasis, scalp psoriasis and having a first-degree relative with PsA reached consensus in round 1 of the Delphi exercise (median >70 mm on a 100-mm VAS), whereas any associated gene (such as HLA-B*08, HLA-B*27, HLA-B*38 or HLA-B*39) reached consensus in round 2 of the Delphi exercise (Supplementary Figure 1).

#### Individuals with psoriasis and asymptomatic synovio-entheseal imaging abnormalities

In round 1 of the Delphi exercise, the terms ‘subclinical PsA’, ‘potential PsA’, ‘psoriasis with imaging findings’, ‘psoriasis with asymptomatic synovio-entheseal imaging findings’ and ‘psoriasis with imaging abnormalities’ were proposed (Supplementary Table 1). Subclinical PsA was generally disliked because the term implies that patients will definitely go on to develop PsA. As consensus was not reached, the three terms with the highest number of votes were moved to round 2 of the Delphi exercise. In round 2, the term ‘psoriasis with asymptomatic synovio-entheseal imaging abnormalities’ outstripped the other terms, gaining almost 60% of the votes. Consensus was finally reached for this term by 85.7% of participants in round 3 of the Delphi exercise. Overall, 86.2% of the participants agreed that this term defines a meaningful population for future research studies.

In round 1 of the Delphi exercise, two definitions were initially proposed for this term (Supplementary Table 2). Consensus was not reached, and panellists suggested two new definitions, which were included in rounds 2 and 3 of the Delphi exercise. Consensus was finally reached in round 3 for ‘any individual with psoriasis and imaging evidence of synovio-entheseal abnormalities that is not associated with clinical signs or symptoms’ (88.6% of the votes).

Imaging modalities that reached consensus for use to define ‘imaging abnormalities’ in round 1 of the Delphi exercise included MRI for axial disease (median on a 100-mm VAS = 97 mm), MRI for peripheral arthritis (86 mm), ultrasonography for peripheral arthritis (90 mm), ultrasonography for enthesitis (90 mm) and plain radiography for peripheral arthritis (70 mm) (Supplementary Figure 2). Participants also voted on which specific MRI and ultrasonography signs should be considered for use in defining imaging abnormalities. MRI signs that met consensus for inclusion were enthesitis (median on a 100-mm VAS = 90 mm), bone marrow oedema (90 mm), synovitis (95 mm), tendonitis (70 mm), bone erosions (81 mm) and new bone formation (80 mm) (Supplementary Figure 3). For ultrasonography, enthesitis (median on a 100-mm VAS = 89 mm), synovitis (97 mm), tendonitis (77 mm) and bone erosions (87 mm) all met consensus in round 1 of the Delphi exercise (Supplementary Figure 4).

#### Individuals with psoriasis and musculoskeletal symptoms not explained by other diagnosis

During round 1 of the Delphi exercise, 93.1% of participants agreed on the term ‘psoriasis with musculoskeletal symptoms not explained by other diagnosis’ (Box 1). In round 2 of the Delphi exercise, 87.9% voted that this term was meaningful for future research studies. Previous iterations of this term included ‘prodromal PsA’, ‘psoriasis with arthralgia’, ‘psoriasis with musculoskeletal symptoms’ and ‘psoriasis with musculoskeletal symptoms without musculoskeletal signs’. Similar to the term ‘subclinical PsA’, participants argued that the term ‘prodromal PsA’ implied that all patients with psoriasis would progress to PsA and was therefore inappropriate.

To define which musculoskeletal symptoms should be considered, participants were provided with a list of symptoms that had previously been identified in the literature as predictors of PsA, along with their hazard ratios and 95% confidence intervals^21^. Participants then scored the factors on a VAS, and a median score of ≥70 mm was defined as reaching consensus. Of these factors, heel pain, stiffness and arthralgia all reached consensus (median scores of 84 mm, 80.5 mm and 75 mm, respectively), whereas fatigue and problems with physical function (median scores of 67.5 mm and 51 mm, respectively) did not.

#### Terms and definitions that did not achieve consensus

Participants were also asked to comment on time points after the diagnosis of PsA, specifically looking at the 6-month and 24-month time points. Although consensus was reached for 6 months from diagnosis being a meaningful time point (90.9%), consensus was not reached on whether this population of individuals should be termed ‘early PsA’, ‘very early PsA’ or ‘new onset PsA’. Similarly, consensus was not reached on whether diagnosis should be defined by satisfying Classification for Psoriatic Arthritis (CASPAR) criteria^22^ and/or musculoskeletal symptom onset. There was a lack of consensus that 24 months was a meaningful time point.

## Future research agenda

During the face-to-face PPACMAN meeting adjacent to the 2019 Annual Meeting of GRAPPA, the results of the consensus exercise were presented, and several areas were identified as priorities for investigation.

### Imaging

Imaging studies (primarily ultrasonography or MRI modalities) have the potential to improve the definition of meaningful subclinical inflammatory states and their ability to predict PsA development. In particular, ultrasonography represents a feasible and adaptable modality that is already being applied in the clinical setting to identify patients with psoriasis who have subclinical enthesitis and/or synovitis^23–25^. High-resolution peripheral quantitative computed tomography (HR-pQCT) also shows promise for use in assessing if the presence of structural entheseal lesions can predict future PsA among patients with psoriasis^26^. Whether sonographic findings (such as synovitis, enthesitis, tenosynovitis or peritonitis) or radiological findings (such as bone erosions, tenosynovitis or bone proliferations) represent abnormal inflammatory features or are simply physiological immune-mediated responses aimed at containing disease progression remains a subject of intense debate. However, reports that the treatment of psoriasis using IL-12–IL-23 blockade^27,28^ or IL-17 blockade^29^ in patients without overt joint symptoms resulted in suppression of these sonographic and radiological abnormalities are promising and will surely aid in the design of future preventive studies, particularly if paired with clinical and molecular risk factor enrichment strategies. These studies will likewise inform a future revision of the term ‘psoriasis with musculoskeletal symptoms not explained by other diagnosis’.

### Prodromal phase and non-specific pain

In an attempt to characterize a ‘preclinical’ phase of PsA, the term ‘psoriasis with musculoskeletal symptoms not explained by other diagnosis’ was selected by stakeholders over ‘prodromal’ and ‘preclinical’, as these terms might imply that the progression from psoriasis to PsA is definite. Indeed, the terms ‘preclinical’ or ‘prodromal’ PsA can only be applied retrospectively at this time owing to a lack of ability to truly predict progression to PsA. Currently, limited data exist with which to conclusively define the non-specific musculoskeletal symptoms that should be considered as part of this phase for research purposes^21^. Further understanding of this stage is crucial to distinguish who might be at the highest risk of progression to PsA and, ultimately, to implement early treatment and prevention strategies for PsA. Information gathered from ongoing^30^ and future longitudinal studies of musculoskeletal symptoms will be needed to further inform and revise this definition.

### Preventive trial design

A preventive medicine approach is not foreign to the field of rheumatology and chronic immune-mediated inflammatory diseases. Specifically, investigators have pioneered trials in preclinical or pre-damage stages of systemic lupus erythematosus and RA, resulting, in some cases, in improved outcomes and even prevention^31–35^. Several prevention trials supported by the US National Institutes of Health are currently underway, including the SMILE study^35^ and the StopRA study^36^, and many other studies are in progress in Europe^37^.

Although the field of psoriatic disease is rapidly moving forward in the design of preventive trials, several questions inevitably remain unanswered and will require a retrospective analytical understanding of the psoriasis-to-PsA transition to be achieved before they can be answered and preventive trials can be initiated. Chief among those questions is how to ascertain the relative weight for each proposed risk factor (in other words, the risk enrichment). The clinical, demographic, genetic and molecular features currently associated with progression from psoriasis to PsA have been mostly derived from retrospective and cross-sectional studies. One strategy for identifying relevant risk factors for progression in prospective studies consists of studying individuals with psoriasis at increased risk of developing PsA who have at least moderate skin disease and imaging evidence of entheseal abnormalities, plus one or more of the following features: scalp involvement, psoriatic nail disease or genetic factors linked to progression (such as a first-degree relative with PsA)^38^.

The specific therapeutic approach in prevention trials will also be challenging, as arguments exist for using medications with any of the available mechanisms of action (such as TNF inhibitors, IL-17 inhibitors, IL-23 inhibitors or phosphodiesterase 4 inhibitors). Importantly, the role of natural history registries (following patients on immunomodulatory therapies as well as those with psoriasis who elect not to be treated with systemic medications) will be of the utmost relevance, as the ultimate goal will be to create a risk-score that incorporates the relative predictive value of each of the proposed risk factors alongside rigorous cut-off thresholds for sensitivity and specificity.

## Conclusions

Given that psoriasis commonly precedes the development of PsA^39^, a unique pre-disease window of opportunity exists in the psoriasis-to-PsA continuum for studies on the clinical and molecular features of transition. To capitalize on this window of opportunity, it is imperative that the preclinical stages of PsA are better understood. The terms and definitions developed by the PAMPA study group describe clinical and imaging features of pre-disease states that individuals might traverse prior to PsA development. The use of standardized nomenclature and common definitions for PsA research will help to facilitate communication and comparison across future studies, which will enable robust validation between efforts in this particularly complex and heterogeneous disease. Overall, it is hoped that the consensus definitions set out in this Consensus Statement will catalyse the development of preventive strategies and, ultimately, improve outcomes in PsA^13^.

Importantly, the overarching output derived from this consensus exercise is to be used exclusively for research purposes at this time. Although necessarily an evolving process, this work represents a much-needed starting point. Furthermore, this terminology should not be viewed as restrictive or unchangeable, nor should it be used for clinical care, given the preliminary nature of these terms and definitions. Adoption of terminology in the clinical sphere will require a natural refinement process and an iterative validation approach as research in this area progresses. Together, these efforts will characterize novel clinical and molecular features associated with the transition of psoriasis to PsA, which in turn will oblige the field to revise the proposed definitions and risk factors.

## Supplementary information

Supplementary Information
