# Supplementary material for: Consensus terminology for preclinical phases of psoriatic arthritis for use in research studies: results from a Delphi consensus study
Source: Nat Rev Rheumatol. 2021 Feb 15;17(4):238–43. doi: 10.1038/s41584-021-00578-2 (PMC7997804; doi:10.1038/s41584-021-00578-2)
Supplement: Supplementary file 1 — Supplementary Information [file 41584_2021_578_MOESM1_ESM.pdf]

nature portfolio

https://doi.org/10.1038/s41584-021-00578-2

Supplementary information

Consensus terminology for preclinical phases of psoriatic arthritis for use in research studies: results from a Delphi consensus study

In the format provided by the authors and unedited

Results from the Pre-Delphi Exercise

At the 2018 PPACMAN annual meeting, we presented stakeholders with preliminary terms and definitions. The presentation consisted of an introductory session where the Preventing Arthritis in a Multi-center Psoriasis At-Risk Population (PAMPA) Study Group was presented, whose aim is to study the clinical, genetic, environmental, and immune events during the natural history of psoriasis into PsA transition. To ultimately achieve this goal, the first unmet need was delineated: consensus definition of an at-risk phenotype(s) based on 1 or more feature. Proposed terms and definitions (shown below in gray boxes) were presented and four breakout workshop sessions allowed for small group discussions in which attendees suggested changes and provided open-ended opinions about the terms and definition proposed. These discussions were followed by a plenary session summarizing each breakout session's outcomes and culminated with a voting exercise utilizing an anonymous automated response system.

Term: "At-risk" for PsA
Definition: *Any individual with psoriasis and one or more risk factor(s) for progression to synovio-entheseal disease*

All small groups commented that "at risk" was not the most appropriate terms as any patient with psoriasis is at risk of progression. Terms such as high risk, higher risk, increased risk, and elevated risk were proposed. In terms of which risk factors should be considered, the groups requested more information regarding the strength of proposed risk factors. It was also acknowledged that this terminology would only apply to those patients who develop skin disease prior to the development of joint pain, which would be a limitation. The group was asked to preliminary vote on the terms that were proposed and results are below:

Voting results:
At risk: 7%
Elevated risk: 38%
High risk: 21%
Increased risk: 34%

→

Increased risk 61%
Elevated risk: 38%

Term: "Subclinical PsA"
Definition: *Any PsO patient with imaging evidence of synovio-entheseal inflammation that is not associated clinical pain or swelling.*

The groups overwhelmingly did not like the term "subclinical PsA" as they thought that this term automatically implied that the patients already have PsA. The groups suggested terms that were more descriptive such as "PsO with asymptomatic enthesopathy", "PsO with positive imaging findings", "subclinical inflammation", "potential PsA", "asymptomatic synovio-entheseal inflammation in patient with PsO". In general, they supported the use of MRI for axial disease and peripheral disease, US for peripheral arthritis, and US for nail disease. For US, they agreed on enthesitis, synovitis, and suggested adding power-doppler. For MRI, they agreed on enthesitis, bone marrow edema, and synovitis. However, erosions/new bone formation in MRI and XR were discussed as to whether these findings alone constituted a diagnosis of PsA. Voting on new proposed terms was done as below:

Voting results:
Psoriasis with imaging findings: 30%
Asymptomatic imaging-based synovio-entheseal psoriasis 13%
Psoriasis with asymptomatic synovio-entheseal inflammation: 47%
Potential PsA: Any PsO patient with imaging evidence of synovio-entheseal *abnormality* that is not associated with clinical pain or swelling and *absence of radiographic changes*: 10%

↓

Psoriasis with imaging findings: 26%
Psoriasis with Asymptomatic synovio-entheseal imaging findings: 19%
Psoriasis with Asymptomatic synovio-entheseal imaging abnormalities: 56%

↓

Psoriasis with imaging findings: 15%
Psoriasis with Asymptomatic synovio-entheseal imaging findings: 15%
Psoriasis with Asymptomatic synovio-entheseal imaging abnormalities: 35%
Psoriasis with imaging abnormalities: 35%

Term: Prodromal PsA
Definition*: PsO patients with arthralgia and/or fatigue without objective evidence of arthritis, enthesitis or dactylitis*

The groups found the term “prodromal” problematic as it insinuates that the patient will always progress to PsA. A more descriptive term, such as “psoriasis with arthralgia and/or fatigue” was proposed as being more appropriate. Groups also commented that musculoskeletal symptoms beyond arthralgia and/or fatigue should be considered. Fatigue itself was also question as it is very non-specific and pain may be confounded by other diseases. An age cut off was also suggested as a possibility given the increased incidence of osteoarthritis in old age. Voting on proposed terms is shown below:

Voting results:
1. Prodromal: 0%
2. Psoriasis with arthralgia: 8%
3. No term/not useful: 29%
4. Psoriasis with MSK symptoms only: 17%
5. Psoriasis with MSK symptoms without MSK signs: 4%
6. Psoriasis with MSK symptoms not explained by other Dx: 42%

↓

1. No term/not useful: 33%
2. Psoriasis with MSK symptoms not explained by other Dx: 67%

How should “new onset” PsA be defined?

The groups were asked how new onset PsA should be defined. Most groups agreed that the use of CASPAR criteria in the definition was most appropriate since it is the classification criteria used in research, but time point who be anchored on musculoskeletal symptom onset. Most groups thought 6 months was an appropriate time point to look at and the term ‘very early PsA’ was suggested. There was no voting on this term.

How should “early onset” PsA be defined?

The groups were also asked how early onset PsA should be defined. There was no consensus on when an appropriate time point. There was no voting on this term.

Terms and Definitions Presented for Voting in the First Delphi Round

Term: At risk for PsA
Definition: Any individual with psoriasis and one or more risk factor(s) for progression to synovio-entheseal disease.

Term: Subclinical PsA
Definition: Any psoriasis patient with imaging evidence of synovio-entheseal inflammation that is not associated with clinical pain or swelling.

Term: Prodromal PsA
Definition: psoriasis patients with arthralgia and/or fatigue without objective evidence of arthritis, enthesitis, spondylitis, or dactylitis or an alternative explanation for musculoskeletal (MSK) symptoms.

Term: 'New onset PsA', defined as fulfillment of CASPAR criteria within the last 6 months
Term: 'Early onset of PsA', defined as fulfillment of CASPAR criteria within the last 24 months

Supplementary Table 1. Voting results for the term "psoriasis with asymptomatic synovio-entheseal imaging abnormalities" and suggested alternatives.

TERMS	Round 1 (Ranked 1 or 2)	Round 2	Round 3
**Subclinical PsA**	17.2%	-	-
**Potential PsA**	3.4%	-	-
**Psoriasis with imaging findings**	27.1%	-	-
**Psoriasis with asymptomatic synovio-entheseal imaging finding**	51.7%	25.0%	11.4%
**Psoriasis with asymptomatic synovio-entheseal imaging abnormalities**	58.6%	59.4%	**85.7%**
**Psoriasis with imaging abnormalities**	41.3%	15.6%	2.9%

Supplementary Table 2. Voting for definitions of term "psoriasis with asymptomatic synovio-entheseal imaging abnormalities".

**DEFINITIONS**	Round 1	Round 2	Round 3
Any psoriasis patient with imaging evidence of synovio-entheseal inflammation that is not associated with clinical pain or swelling	34.5%	12.5%	NA
Any psoriasis patient with imaging evidence of synovio-entheseal abnormalities that is not associated with clinical pain or swelling and in the absence of radiographic changes.	58.6%	21.9%	0%
Any psoriasis patient with imaging abnormalities and absence of musculoskeletal symptoms	NA	18.8%	11.4%
Any psoriasis patient with imaging evidence of synovio-entheseal abnormalities that is not associated with clinical signs or symptoms	NA	46.9%	**88.6%**

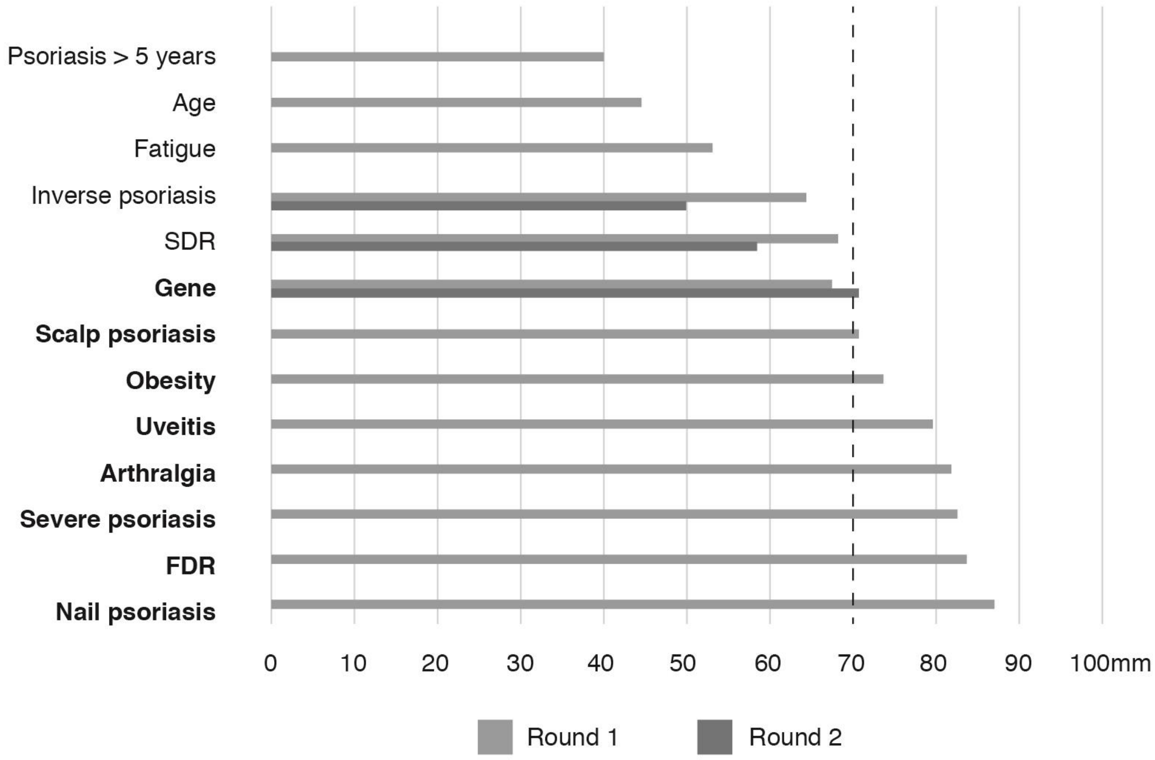

Supplementary Figure 1. **Risk factors to define "increased risk for PsA".**
Results of voting on risk factors in Rounds 1 and 2. Risk factor medians ≥ 70 (dashed line) indicate that they met consensus. SDR. Second degree relative; FDR, first degree relative.

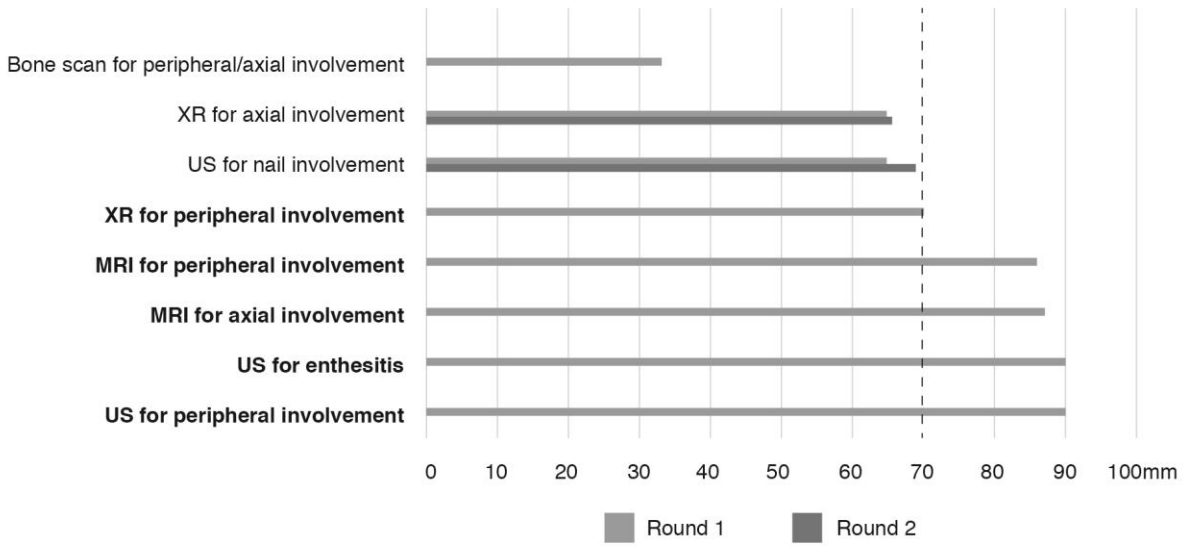

Supplementary Figure 2. Imaging modalities used to describe "imaging abnormalities".
Results of voting on imaging modalities in Rounds 1 and 2. Medians ≥ 70 (dashed line) indicate that they met consensus. XR, X-ray; US, ultrasound; MRI, magnetic resonance imaging.

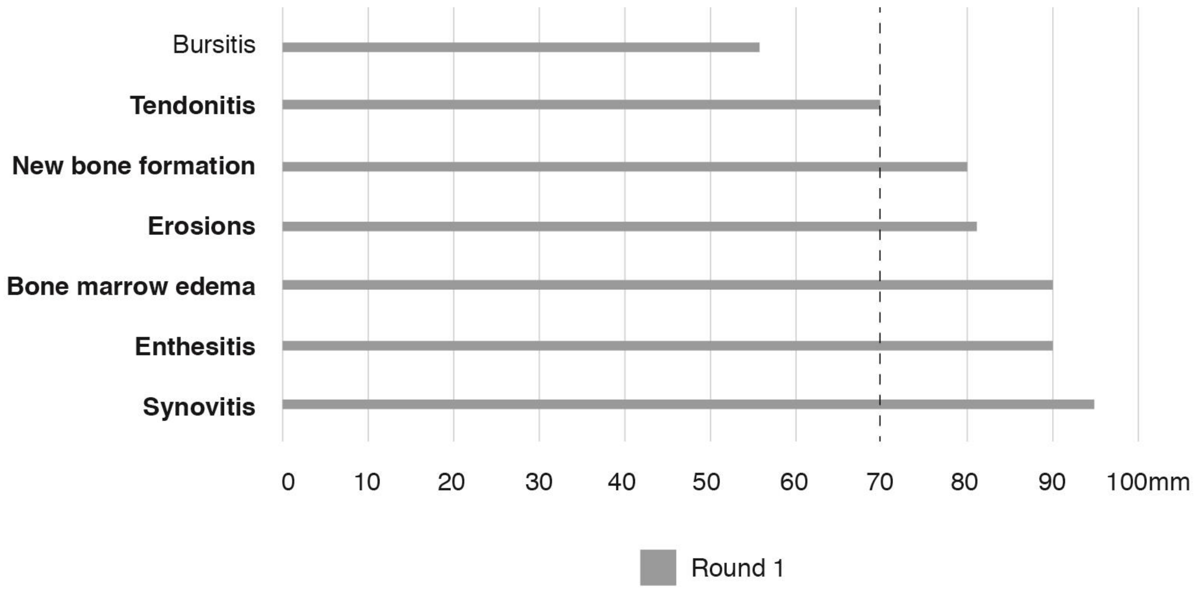

Supplementary Figure 3. Magnetic resonance imaging signs on imaging to signify abnormalities. All results from Round 1. Medians ≥ 70 (dashed line) indicate that they met consensus.

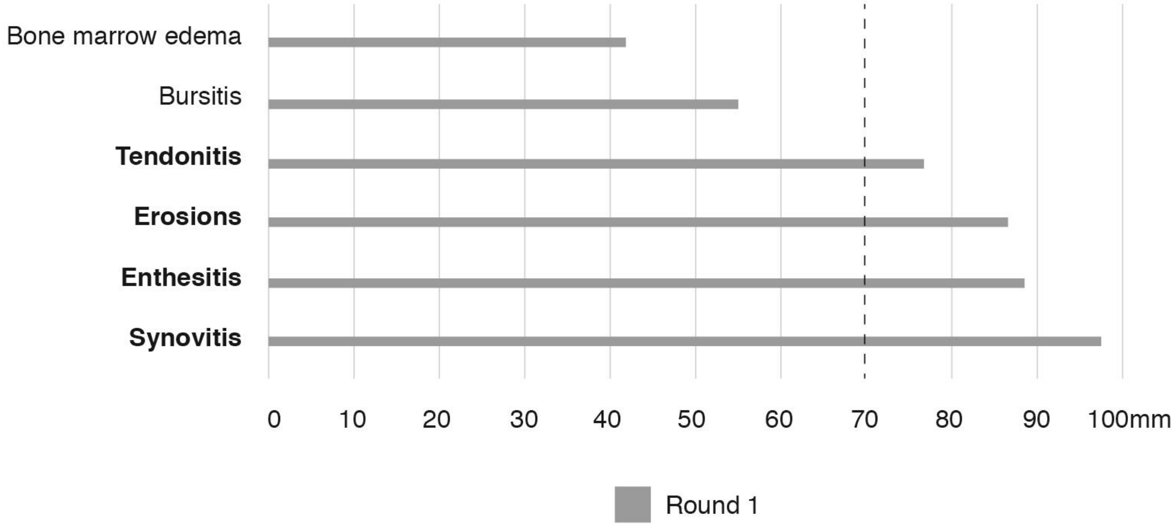

Supplementary Figure 4. Ultrasound signs on imaging to signify abnormalities.
All results from Round 1. Medians ≥ 70 (dashed line) indicate that they meet consensus.
